# Randomized trial of exercise in sedentary middle aged women: effects on quality of life

**DOI:** 10.1186/1479-5868-3-34

**Published:** 2006-10-04

**Authors:** Deborah J Bowen, Megan D Fesinmeyer, Yutaka Yasui, Shelley Tworoger, Cornelia M Ulrich, Melinda L Irwin, Rebecca E Rudolph, Kristin L LaCroix, Robert R Schwartz, Anne McTiernan

**Affiliations:** 1 Cancer Prevention, Fred Hutchinson Cancer Research Center, 1100 Fairview Ave N, Seattle WA 98109, USA; 2 Department of Public Health Sciences, University of Alberta, 13-106J Clinical Sciences Building, Edmonton, AB, 6G 2G3, Canada; 3 Channing Laboratory, Department of Medicine, Brigham and Women's Hospital and Harvard University, 181 Longwood Avenue, Boston MA 02115, USA; 4 Department of Epidemiology and Public Health, Yale University, PO Box 208034, New Haven CT 06520, USA; 5 Health Services Research and Development Center of Excellence, Department of Veterans Affairs, 1100 Olive Way, Suite 1400, Seattle, WA, 98101, USA; 6 Department of Internal Medicine, University of Colorado Health Sciences Center, 4200 E. 9th Avenue, Campus Box B-179, Denver CO 80262, USA

## Abstract

Increasing physical activity is currently considered to be a possible prevention strategy for cancer, obesity, and cardiovascular disease, either alone or in combination with dietary changes. This paper presents results of a randomized trial of moderate-to-vigorous intensity exercise in middle aged, sedentary women; specifically, we report changes in and correlates of quality of life and functional status of this exercise intervention program for both the short (three months) and longer term (12 months). The intervention group showed a significant increase in Mental Health score from baseline to 3 months (p < .01), significantly greater than the change in the control group at 3 months (p < .01). A similar trend among exercisers was observed for the General Health score (p < .01), and this finding was significantly greater than the change in control group at 3 months (p = .01). Change in Social Support – Affection were predictors of the changes in quality of life variables. This study documented improvements in quality of life and general functioning that occurred as a result of participating in an exercise intervention in sedentary middle-aged women.

## Background

Physical activity increase is currently under study as a possible prevention strategy for cancer, obesity, and cardiovascular disease [1-3], either alone or in combination with dietary changes. The level of physical activity needed to alter chronic disease patterns is currently the subject of debate. The Healthy People 2010 goals include increasing the number of people that are moderately physically active (e.g., walking) five or more times per week for 30 minutes per day [4]. These general population goals are reasonable and have been related to cardiovascular fitness, but they may not be intensive enough to reduce obesity-related health problems and to ultimately affect cancer risks; therefore, more rigorous goals have been proposed and used in recent studies [5,6]. The Institute of Medicine, for example, recommends an hour per day of moderate to vigorous exercise 4–7 days per week [7].

This level of exercise has the potential to alter the ways in which people think, feel, and conduct their lives on multiple levels. These effects could most likely to be seen in sedentary people who undertake a regular exercise program. In general, experimental studies of exercise have found improvements in various aspects of quality of life [8] including general functioning [9-12] and depression and anxiety symptoms [13-15]. Most of these studies have found positive effects of exercise on multiple aspects of quality of life and functional status during the active intervention period or at relatively short-term intervention follow-up periods of 4 – 20 weeks. The mechanisms by which these improvements occur are not known, but hypotheses include improvements in psychological factors, such as body image or perceptions of physical fitness, as well as more physiological changes, such as sympathetic nervous system activity. Long-term quality of life effects due to large increases in exercise are unknown. Further, if increases in quality of life and functional status remain after the initiation of intervention they could contribute to long-term adherence.

However, it is also possible that intensive exercise could produce negative effects on relevant aspects of quality of life, especially in initially sedentary individuals. Possible negative effects include body or joint pain, disruption of social interactions, or negative effects on mood from the high demands of the exercise program. Little is available in the literature documenting any of these negative effects. These deleterious effects may be short-lived and may decrease over time, or they may only occur in the long run, after the initial short-term behavior changes have occurred. As with the long-term causes of improvements in functioning due to exercise, little is known about the mechanisms of any negative effects of exercise on general functioning.

We conducted a randomized trial of moderate-to-vigorous exercise in post-menopausal, sedentary women to determine the effects of increased activity on blood hormones and on adiposity. The effects of this intervention on multiple biological and health outcomes have been published elsewhere [5,16,17]. Briefly, women achieved and maintained high levels of exercise in the intervention group, compared with controls, over a 12-month period. The present study builds on these findings by reporting changes in and correlates of quality of life of this intensive exercise intervention program for both the short (three months) and longer term (12 months).

## Experimental methods

Details of the aims, experimental design, and measurement protocols of the Physical Activity for Total Health Study have been published previously [6,18]. Briefly, the study was a randomized controlled intervention trial comparing the effects of a one-year moderate-to-vigorous intensity aerobic exercise intervention on body fat and sex hormone concentrations measured at three and 12 months after study randomization, all compared to a stretching control group. The exercise intervention was designed to be a combined exercise intervention package: short-term (3 months post-randomization) intensively monitored exercise program at a facility, followed by a longer-term (until 12 months post-randomization) program primarily occurring at participants' homes.

### Participants

Participants were postmenopausal women from the greater Seattle area who were ages 50 to 75 years at entry, sedentary at baseline (< 60 mins/week of moderate- or vigorous-intensity recreational activity and a maximal oxygen consumption (VO_2_max) < 25.0 ml/kg/min), with a BMI ≥ 25.0 kg/m^2 ^(or a BMI between 24.0 and 25.0 if percent body fat > 33.0), not taking hormone replacement therapy, no clinical diagnosis of diabetes and fasting glucose levels < 140 mg/dL, and non-smokers.

Women were recruited through a combination of mass mailings and media placements. Details on recruitment have been published elsewhere [18]. After extensive screening, 173 women were eligible and participated in the trial. They were randomly assigned to the aerobic exercise intervention (n = 87) or the control group (n = 86). Randomization was stratified by BMI (< 27.5 kg/m^2 ^vs. ≥ 27.5 kg/m^2^) to ensure balanced numbers of heavier and lighter women in each study arm.

### Exercise intervention

The exercise prescription consisted of at least 45 minutes of moderate-intensity aerobic exercise 5 days per week for 12 months [19]. A combined exercise facility and home exercise program was offered. Participants were required to attend 3 sessions per week at the facility during months 1, 2, and 3, and to exercise 2 days per week at home. For months 4 through 12, participants were required to attend at least 1 session/week at the facility (participants were allowed to exercise additional days at the facility if they chose) and to exercise the remaining days on their own for a total of 5 days per week. The training program started at 40% of VO_2_max for 16 mins/session and gradually increased to 60 – 75% of VO_2_max for 45 min session by week 8, where it was maintained for the duration of the study. Participants wore Polar heart rate monitors during their exercise sessions. Facility sessions consisted of treadmill walking and stationary bicycling. Participants also did approximately 5 – 10 minutes of light weight training 3 days per week to strengthen muscles around joints to help reduce risk of injury and to improve adherence to the aerobic exercise intervention. A variety of home exercises were suggested and encouraged, including walking, aerobics, and bicycling. Participants were also encouraged to wear their heart rate monitors when exercising at home.

Several techniques for promoting adherence were used including: 1) individualized attention in facility classes; 2) individual and group exercise behavior-change education classes; 3) weekly phone calls to promote adherence for non-adherent participants; 4) individual meetings at baseline and every 3 months to outline goals and provide feedback on progress; 5) incentives such as water bottles and other study items; 6) quarterly newsletters; and 7) tri-annual intervention group activities such as hikes.

Women randomized to the control group attended weekly 45-min stretching sessions for the entire year-long study period, and were asked not to change other exercise habits during the study. All exercisers and stretching control participants were asked to eat their usual diet during the yearlong study.

### Measurement strategy

Study participants provided data on a variety of outcome variables collected at baseline, 3-month, and 12-month follow-up time points. We selected the main outcome variables listed below because of their relevance to overall functioning, their well known measurement properties, and the existence of normative data. We selected additional independent or process variables for two reasons: they were hypothesized to improve as a result of the intervention and therefore improve quality of life (e.g., feelings about body) or they were hypothesized to worsen as a result of the intervention and thereby affect quality of life and general functioning (e.g., pain symptoms, perceived stress, and social support).

### Main outcome variables

#### Mental, physical, and general health

The SF-36 Health Survey is commonly used to evaluate an individual's basic functioning on several scales. We used the Mental Health, Physical Health, and General Health multi-item scales from the SF-36 Health Survey [20]. The Mental Health scale is composed of items such as "Have you been a very nervous person?" and "Have you felt calm and peaceful?" Participants selected their responses on a six-point Likert scale from 1 (none of the time) to 6 (all of the time). The General Health scale is composed of items such as "I am as healthy as anyone I know." and "I expect my health to get worse." Participants selected their responses on a five-point Likert scale from 1 (definitely false) to 5 (definitely true). The Physical Health scale is composed of items such as "Does your health currently limit you in climbing several flights of stairs?" and "Does your health currently limit you in bending, kneeling, or stooping?" Participants selected their responses on three-point Likert scale from 1 (yes, limited a lot) to 3 (no, not limited at all). All three scales were scored according to guidelines described in the SF-36 Health Survey Manual and Interpretation Guide [21].

#### Emotional symptoms

We used subscales of the Brief Symptom Inventory (BSI) to measure anxiety, and we used a modified version of the BSI to measure depression [22]. The survey questions composing each subscale were rated on a five-point Likert scale from 1 (not at all) to 5 (extremely). The Anxiety subscale consists of six questions including how often the respondent experienced "nervousness or shakiness inside" or "feeling fearful" over the past seven days. The modified Depression subscale consists of six questions including how often the respondent experienced "feeling lonely" or "feeling hopeless about the future" over the past seven days.

### Process variables

#### Social support

The MOS Social Support Survey is a validated scale used to measure 19 aspects of functional social support [23]. We used a modified version of the MOS Social Support Survey to measure four social support outcomes (emotional/informational, affection, tangible, and overall). All component variables of the Social Support Survey are measured on a five-point Likert scale from 1 (none of the time) to 5 (all of the time). The modified Emotional/Informational scale is composed of three questions asking how often the respondent has "Someone you can count on to listen to you when you need to talk," "Someone to give you good advice about a problem," and "Someone to share your most private worries and fears." The modified Affection scale is composed of one question asking how often the respondent has "Someone to love you and make you feel wanted." The modified Tangible scale is composed of two questions asking how often the respondent has "Someone to take you to the doctor if you need it," and "Someone to help with daily chores if you are sick." The modified Overall scale is composed of all six previously described questions, as well as an additional question asking how often the respondent has "Someone to do something fun with."

#### Perceived stress

The Perceived Stress Scale measures an individual's self-perceived stress response to events occurring over the past month [24]. We employed a four-item brief form of the 14-item survey instrument for this study. The brief form of the scale is composed of four questions which respondents answered using a five-point Likert scale from 1 (never) to 5 (very often). The scale is composed of questions such as how often respondents "Felt things were going your way" and "Felt you were unable to control important things in your life."

#### Exercise adherence

Daily activity logs were completed every day by intervention participants and turned in weekly to the exercise trainer. Only sports or recreational activities of at least a 3 MET level (based on the Compendium of Physical Activities [25]) were included in the analyses. Duration (minutes/week) was summed for the entire year with the average minutes per week used as a measure of adherence. We defined good adherence as meeting 80% of the exercise prescription (45 minutes, 5 times per week of moderate-to-vigorous intensity sports/recreational aerobic exercise). Measures of adherence included: duration (minutes per week of exercise), intensity (% Heart Rate Reserve (%HRR) = (HR during exercise – Resting HR)/HRmax – Resting HR), dose (kcal/week = duration*intensity* VO_2_max *5 kcal/L O_2_* 1 L/1000 mL* kg body weight). When determining %HRR, resting HR and HRmax from the baseline VO_2_max treadmill test were used. If a HR value was not recorded in the daily activity log for a particular activity, a HR value was assigned (e.g., if the activity was walking and a HR value was available for that person from another day, then that HR value would be assigned).

#### Cardiorespiratory fitness

Maximal oxygen consumption (VO_2_max) was assessed at baseline and 12 months. All participants completed a maximal-graded treadmill test, with heart rate and oxygen uptake monitored by a Medgraphics automated metabolic cart (Medgraphics, MN).

#### Knee pain

As part of a symptom checklist, we asked participants if they experienced pain or discomfort in their knees, with responses from not at all (1) to yes and it bothers me very much (4). The response format was modeled after the Women's Health Initiative symptom checklist [26,27]. For comparison purposes, the responses were transformed into a score from 0 – 100, where 0 represented the highest pain and 100 represented no pain.

### Demographic variable

We measured key demographic variables such as age, ethnicity, education, and marital status using single item questions.

### Data analysis

All of the analyses were based on assigned intervention at the time of randomization, regardless of adherence or compliance status (i.e., intent to treat analysis). First, we assessed changes in the outcome variables from baseline to 3-month, and 3-month to 12-month follow-up and their differences for intervention and comparison participants. We then tested for differences from baseline to follow-up in the process variables. Finally, among intervention participants only, we regressed change in selected outcome variables in the short-term (3 months) and long-term (12 months) follow-up on a set of independent variables, including adherence to study exercise goals, changes in fitness, and changes in selected process variables. We selected the Mental Health, Physical Health, and General Health outcome variables for this intervention-only analysis based on our finding of significant improvements in these variables in the intervention group. We used the significance results of Table 2 to include two possible mechanism variables: Social Support – Affection and knee pain. To account for the longitudinal nature of the data, we used a generalized-estimating-equation modification of the linear regression model [28].

**Table 1:** Changes in Quality of Life for Intervention and Control Participants in an Intensive Exercise Intervention

	Baseline	3-month follow up		12-month follow up	
	Unadjusted (std. dev.)	Unadjusted (std. dev.)	P^1^	Unadjusted (std. dev.)	P^1^
**Mental health†**					

Between Groups			**< 0.01**		0.15
Within Intervention (N = 86)	80.28 (15.21)	85.20 (11.03)	**<0.01**	83.27 (12.67)	**0.08**
Within Control (N = 85)	81.46 (11.95)	81.24 (12.32)	0.72	81.84 (11.51)	0.78
**Anxiety**					
Between Groups			0.93		0.50
Within Intervention (N = 87)	94.49 (11.45)	95.31 (9.67)	0.28	94.36 (10.94)	0.99
Within Control (N = 86)	94.08 (7.41)	94.79 (9.37)	0.48	95.09 (8.16)	0.48
**Depression**					
Between Groups			0.65		0.49
Within Intervention (N = 86)	93.56 (11.19)	94.88 (7.93)	0.25	94.31 (10.40)	0.38
Within Control (N = 86)	91.96 (9.63)	92.61 (8.52)	0.51	93.45 (8.03)	**0.05**
**General health**†					
Between Groups			**<0.01**		**0.02**
Within Intervention (N = 87)	79.95 (14.88)	86.12 (12.50)	**<0.01**	83.55 (13.56)	**<0.01**
Within Control (N = 86)	79.52 (11.83)	80.67 (13.41)	0.27	78.74 (14.08)	0.47
**Physical functioning**†					
Between Groups			**0.07**		**<0.01**
Within Intervention (N = 87)	85.86 (14.45)	87.41 (13.25)	0.28	88.60 (14.24)	0.13
Within Control (N = 86)	86.40 (11.55)	83.99 (16.07)	0.14	83.18 (15.49)	**0.02**

^1^P-values for each outcome correspond to (top to bottom) between groups differences and within groups change from the previous time point.

†Higher scores indicate better state of being

**Table 2:** Changes in Process Variables for Intervention and Comparison Participants in an Intensive Randomized Exercise Intervention Trial

	Baseline	3-month follow up		12-month follow up	
	Unadjusted (std. dev.)	Unadjusted (std. dev.)	P^1^	Unadjusted (std. dev.)	P^1^
**Perceived stress**					
Between Groups			0.98		0.36
Within Intervention (N = 87)	79.38 (16.95)	78.59 (17.04)	0.63	78.13 (18.20)	0.53
Within Control (N = 86)	78.42 (16.03)	77.53 (15.28)	0.64	79.39 (16.02)	0.50
**Social support – overall**					
Between Groups			0.23		0.37
Within Intervention (N = 87)	80.26 (19.83)	78.74 (21.46)	0.37	79.49 (20.78)	0.72
Within Control (N = 86)	75.62 (23.94)	76.70 (20.03)	0.43	77.08 (20.08)	0.35
**Social support – tangible**					
Between Groups			0.30		0.40
Within Intervention (N = 87)	80.03 (25.89)	76.72 (26.49)	0.17	79.42 (25.64)	0.79
Within Control (N = 86)	74.71 (27.98)	74.55 (25.41)	0.93	76.64 (24.45)	0.36
**Social support – affection**					
Between Groups			**0.06**		**0.04**
Within Intervention (N = 86)	83.14 (26.41)	80.75 (27.41)	0.46	79.88 (27.90)	0.29
Within Control (N = 86)	79.65 (28.57)	84.23 (23.85)	**0.04**	84.23 (23.54)	**0.05**
**Social support – emotional**					
Between Groups			0.63		0.97
Within Intervention (N = 87)	79.41 (23.11)	78.54 (22.79)	0.65	79.47 (23.28)	0.86
Within Control (N = 86)	74.13 (27.86)	74.31 (24.92)	0.81	74.60 (25.21)	0.79
**Knee pain**					
Between Groups			0.50		0.91
Within Intervention (N = 86)	85.27 (20.84)	78.16 (27.76)	**<0.01**	76.83 (29.49)	**0.02**
Within Control (N = 86)	81.78 (23.24)	76.98 (28.33)	0.11	73.41 (32.22)	**0.01**

^1^P-values for each variable correspond to (top to bottom) between groups differences and within groups change from the previous timepoint.

†Higher scores indicate better state of being (e.g., more social support, less pain)

## Results

All 173 randomized women provided responses to survey questions regarding quality of life and psychosocial characteristics at baseline. Complete survey responses were available for 171 women at 3 months and for 166 women at 12 months. We included data from several women who did not complete the full survey, yet did provide sufficient data to be included in portions of the analyses. We detected no pattern of omission among these partially completed surveys. As a result of including these data, sample sizes vary slightly between several of our analyses. Fitness measurements were available for 172 women at baseline and for 157 women at 12 months. Analyses of fitness measures only include the 157 women for whom we had complete fitness data at both time points.

Demographic variables were similar between the intervention and control groups. Participants on average were aged 61 years and highly educated (91% were high school graduates). Less than a third of the participants worked full-time, and 86% were non-Hispanic White, 4% were African-American, and 6% were Asian American.

Group-specific mean values for quality of life variables (Mental Health, Anxiety, Depression, General Health, Physical Functioning) were not significantly different between the intervention and control groups at baseline (Table 1). The intervention group showed a significant improvement in Mental Health from baseline to 3 months (p < .01), and this change was significantly higher than the change in control group at 3 months (p < .01). Despite an insignificant between-groups difference in Mental Health at 12 months, the intervention group showed a marginally significant improvement in Mental Health from baseline to 12 months (p = .08). Similar results were observed for the General Health variable. The intervention group showed a significant improvement in General Health from baseline to 3 months (p < .01), and this change was significantly higher than the change in control group at 3 months (p < .01). The between-groups difference was significant at 12 months and the intervention group also showed a significant improvement in General Health from baseline to 12 months (p < .01). The control group showed a significant improvement in Depression from baseline to 12 months (p = .05); no significant difference was observed in the intervention group.

Group-specific mean values for process variables (Table 2; Perceived Stress, Social Support – Overall, Social Support – Tangible, Social Support – Affection, Social Support – Emotional/Informational and knee pain) were not significantly different between the intervention and control groups at baseline. The control group showed a significant improvement in Social Support – Affection from baseline to 3 months (p = .04), and had marginally significantly higher Social Support – Affection than the intervention group at 3 months (p = .06). The control group also had a significant improvement in Social Support – Affection from baseline to 12 months (p = .05), which was significantly higher than the improvement in the intervention group (p = .04). Knee pain worsened significantly from baseline to 3 months in intervention women and from baseline to 12 months in both intervention and control women. There were no significant between-groups or within-group differences for the remaining variables at any other time points.

The results of the intervention-only analyses in exercisers are presented in Tables 3, 4, and 5. Change in Social Support – Affection Health was significantly associated with changes in Mental Health from baseline to 3 months (p < .01) and from baseline to 12 months (p < .01). No other variables were significantly associated with changes in Mental Health scores for intervention women. Change in Social Support – Affection showed similar associations with changes in General Health from baseline to 3 months (p = .01) but not from baseline to 12 months. Changes in knee pain were associated with changes in perceptions of General Health with marginal significance (p = .07). Finally, increases in physical fitness were associated with increases in Physical Functioning scores from baseline to 12 months (p = .01), and increase in Social Support – Affection was marginally associated with changes in Physical Functioning from baseline to 3 months (p = .10). No other variables significantly predicted Physical Functioning.

**Table 3:** Predictors of Mental Health Scores in Intervention Women at Short- and Long-Term Follow-ups*

	Change from baseline to 3 months		Change from baseline to 12 months	
	β	P	β	P
Adherence	0.00	0.62	0.02	0.27
Change in Fitness (VO_2_max)	NM	-	-0.45	0.33
Social Support – Affection	**0.15**	**< 0.01**	**0.19**	**< 0.01**
Knee Pain	-0.04	0.21	0.04	0.40

*Adjusted for baseline mental health

NM = not measured

**Table 4:** Predictors of General Health Scores in Intervention Women at Short- and Long-Term Follow ups*

	Change from baseline to 3 months		Change from baseline to 12 months	
	β	P	β	P
Adherence	0.01	0.22	0.01	0.40
Change in Fitness (VO_2_max)	NM	-	0.58	0.23
Social Support – Affection	**0.11**	**0.01**	0.04	0.46
Knee Pain	0.02	0.63	**0.09**	**0.07**

* Adjusted for baseline general health

NM = not measured

**Table 5:** Predictors of Physical Functioning Scores in Intervention Women at Short- and Long-Term Follow-ups

	Change from baseline to 3 months		Change from baseline to 12 months	
	β	P	β	P
Adherence	0.01	0.51	0.01	0.76
Change in Fitness (VO_2_max)	NM	-	**1.54**	**0.01**
Social Support – Affection	0.08	0.10	-0.03	0.54
Knee Pain	0.03	0.46	0.06	0.28

* Adjusted for baseline physical functioning

NM = not measured

## Discussion

This study documented changes in quality of life and general functioning that occurred as a result of participating in an intensive exercise intervention in sedentary middle-aged women. In accordance with our hypotheses, several aspects of the intervention participants' quality of life significantly improved compared to control participants. These improvements occurred in the areas of mental health, general health perceptions, and physical functioning, representing diverse aspects of functioning. These changes were most pronounced at the 3-month follow-up. They persisted at the 12-month follow-up for general health perceptions and to a lesser extent for physical and mental health values. These findings are concordant with the literature from other randomized trials in which quality of life and functioning also improved [11]. Overall, participating in an intensive exercise intervention is a positive choice for sedentary women.

The values reported at baseline for the Physical Functioning, Mental Health, and General Health subscales of the SF-36 were slightly higher than reported national norms [20]. The national norms for the three subscales were 82.86, 74.36, and 70.48, respectively, for females aged 45 – 54, while our baseline values were 4 – 9 scales points higher, depending on the scale. We would expect these higher levels of functioning, given the rigorous recruitment methods used in this study, screening for ability to complete the study and participate fully in all aspects of research and intervention requirements. We found in a post-hoc analysis that baseline values were moderately correlated with changes in the respective variable at 3 month follow-up (correlations ranged from .38–.56; p < .01 for all). However, even in this conservative situation, exercise improved quality of life. One might expect even larger levels of improvement in studies where the recruitment process was not so stringent. Therefore, exercise might be even more of a benefit to women from the general population. Conversely, women from a more general population sample might be less likely to adhere to the study protocol and therefore experience fewer benefits.

These results suggest that participation in the control group of this study produced improvements in depression. The control group participated in a stretching/relaxation program for the year-long period of intervention; therefore, this improvement may reflect a real improvement due to the stretching activities conducted in a group setting. We previously reported that women in the control group had improvements in self-reported sleep quality [17]. In fact, the idea that stretching might have improved an emotional aspect of quality of life makes this study design relatively conservative, in that the intervention may have been simply protective against decreases over time, but we would not be able to detect this protection because of improvements in the control group. This lends strength to the argument that the exercise intervention did improve quality of life. The exercise effects observed in this study must be understood as those over and above the stretching/relaxation effects of the control group. Physical functioning decreased over time in the controls, and therefore it seems as though the intervention protected against gradual physical decline.

Few of the potential negative effects hypothesized to occur as a result of intervention participation were documented in this study. Intervention women, as compared to control women, did not report increased stress or general or tangible social support. These types of changes would have likely been identified at the 3-month follow-up, when intervention participants were at the most intensive level of participation. Of course, it is possible that other negative outcomes, not measured in this study, may have occurred and could have interfered with the participants' lives or exercise habits. However, the measures used in this study represent a broad array of symptoms and problems that could occur as a result of the intervention. Therefore, we feel reasonably sure that the intervention did not cause many negative effects on women's lives.

The exceptions to the "no harm" finding were in the increases in knee pain and decreases in affection-oriented social support that women reported as a result of the intervention. This is not surprising because sedentary women became regular exercisers and adhered to the intensive study protocol within three months. Even experienced exercisers develop pain in lower joints that can interfere with exercise and movement. This increase of knee pain was about the same size as the improvements in other more general functioning variables. Therefore, decrements in symptoms were potentially balanced with improvements in other aspects of functioning. Because control women also reported increases in knee pain, it is possible that these reports for both groups reflect normal changes over time.

We also attempted to identify mechanisms through which exercise may change quality of life. High levels of social support (specifically the subscale on feeling affection from others) predicted high levels of Mental Health scores, predicted General Health changes at three months, and were borderline predictors of Physical Functioning changes at three months. Even though there were no significant increases in social support from baseline to follow-up at 3 or 12 months, it is possible that social support and mental health levels are related in this way, in a subset of participants. This suggests that exercising might attract positive social comments and interactions, especially early in the change process. There is some support from the literature on this finding, as making dramatic dietary changes also engenders positive comments from family and friends [29]. Thus, engaging and successfully completing healthy behavior programs may be an admirable and positive social cue for others.

There are several limitations to the present study. First, the participants were carefully screened before the study for their ability to perform the tasks of the research project, including making intensive exercise changes. These women were willing to and possessed the resources and interest to make large changes in exercise and to maintain them over the course of the one-year intervention period. These factors limit the generalizability of the findings. Also, the participants in this study reported higher functioning at baseline compared to the general population. Therefore any improvements in quality of life may be muted because of high baseline values, but despite this we saw improvements in quality of life due to exercise. Alternatively, negative effects of exercise could have been blunted due to better compensation in these well-resourced women. The exercise intervention was carefully delivered and supervised, including multiple visits per week to the exercise facility. Therefore, women had expert support for making and maintaining injury-free behavioral changes. We may see different patterns of findings in settings where women begin and maintain this type of exercise without such support. We can only report on possible improvements and decrements that were measured, not on those that we did not identify. Therefore, it is possible that there are other issues important to women that we are not able to examine. Adherence to the study protocol was relatively high, and so we might not have enough variance to find any sort of potential dose-response relationship of adherence to quality of life and functioning. This limits the extent of the knowledge of a potential threshold effect in exercise's effects on functioning. Finally, the control group improved its quality of life, and therefore we might be underestimating the effects of exercise by comparing it to the improved control functioning.

Strengths of this study include its randomized design and careful intervention delivery. Careful measurement of adherence to the study protocol allowed us to verify self-reports of exercise. This suggests that over-reporting due to social desirability was minimized.

## Conclusion

In conclusion, we found that a moderate to intensive exercise intervention had some positive and no negative effects on functioning. Improvements occurred in physical functioning and in other areas of functioning. Support and physical fitness played a role in these functioning changes. This is good news for exercise programs, in that positive effects on mental and physical health could support long term adherence to the exercise regime.

Future research needs clearly come from these findings. Studies should be conducted in public health settings to determine if different populations report the same types of changes and improvements. To affect public health improvements in functioning and disease risk, we hope that all women, regardless of baseline functioning, will engage in increases in exercise. For such behavioral changes to occur, they must be relatively pain and discomfort-free and must fit within women's diverse lifestyles and support structures. Determining the effects of exercise in the population is one of the next steps to using exercise and physical activity to improve the public's health.
